# Acircadian rhythm-related gene signature for predicting survival and drug response in HNSC

**DOI:** 10.3389/fimmu.2022.1029676

**Published:** 2022-11-24

**Authors:** Chuan Zhang, Dan Dang, Hongrui Wang, Shuyou Shi, Jiayu Dai, Ming Yang

**Affiliations:** ^1^ Department of Pediatric Surgery, First Affiliated Hospital of Jilin University, Changchun, China; ^2^ Department of Neonatology, The First Hospital of Jilin university, Changchun, China; ^3^ Department of Molecular Biology, College of Basic Medical Sciences, Jilin University, Changchun, Jilin, China; ^4^ College of Clinical Medicine, Jilin University, Changchun, Jilin, China

**Keywords:** circadian rhythm, prognosis, head and neck squamous cell carcinoma, drug response, gene signature

## Abstract

Head and neck squamous cell carcinoma (HNSC) represents one of the most common malignant carcinomas worldwide. Because the 5-year survival rate of patients with HNSC is poor, it is necessary to develop an effective signature for predicting the risk of HNSC. To identify a circadian rhythm (CR)-related predictive signature, we analyzed the RNA-seq data of patients with HNSC from The Cancer Genome Atlas and Gene Expression Omnibus cohorts. Nine CR-related genes (*PER2*, *PER3*, *GHRL*, *CSF2*, *HDAC3*, *KLF10*, *PRKAA2*, *PTGDS*, and *RORB*) were identified to develop a CR-related signature. The area under the curve values for 5-year overall survival were 0.681, 0.700, and 0.729 in the training set, validation set, and an external independent test set (GSE41613), respectively. The Kaplan‒Meier curve analysis showed that the high-risk group had a reduced relapse-free survival compared with the low-risk group in the training set, validation set, and test set (*P* < 0.05). Finally, we observed that the CR-related gene signature was associated with the tumor immune microenvironment, somatic nucleotide variation, and drug response in HNSC. In conclusion, we developed a circadian rhythm-related gene signature for predicting overall survival in HNSC.

## Introduction

Head and neck squamous cell carcinoma (HNSC) represents one of the most common malignant carcinomas worldwide (1) and is characterized by heterogeneity and aggressiveness. Despite substantial efforts invested into the therapeutic development of HNSC, the 5-year survival rate of patients with HNSC remains poor (2). Therefore, it is clinically necessary to identify a comparatively reliable and applicable prognostic signature for HNSC to guide clinical decision-making. Several gene signatures have been developed to predict the prognosis of HNSC (3–5). However, due to the heterogeneity of HNSC, the predictive ability of these indicators is not satisfactory. Therefore, identifying a novel biomarker for predicting overall survival for HNSC is urgent.

Circadian rhythms are 24-h oscillations that affect multiple biological functions in humans (6). Circadian rhythm disorders are linked to aggressive tumor behaviors and unwanted clinical outcomes. Circadian-related genes have been implicated in the pathogenesis of colorectal cancer (6), prostate cancer (7), and bladder cancer (8). Moreover, emerging evidence suggests the involvement of circadian rhythm in the tumor immune microenvironment (9–11). Because the tumor immune microenvironment has an important influence on the effect of tumor immunotherapy, the circadian rhythm may affect the sensitivity of immunotherapy by affecting the immune microenvironment.

While circadian rhythm has recently become a hot topic in the cancer research field, the specific mechanisms of its role in humans are still not fully understood. Specifically, the impacts of circadian rhythm disruption on the prognosis of HNSC and the immunotherapeutic effect remain unclear. Considering the involvement of circadian rhythm disturbance in aggressive tumor behaviors and unwanted clinical outcomes in several types of cancer, we hypothesized that a circadian rhythm (CR)-related gene signature may be used to predict prognosis and immunotherapeutic effects in HNSC patients.

Human papillomavirus (HPV) has emerged as a reliable predictor for the progression of HNSC (12). In addition, HPV infection has been directly linked to a higher morbidity of oropharyngeal cancer in men under 50 who do not smoke or drink (13). HPV infection affects the mutational landscape and correlates with an improved prognosis (14). Therefore, a hypothesis has been proposed that HPV infection may be involved in gene expression regulation (14). In the present study, we investigated whether circadian rhythm disruption is related to HPV infection.

To establish a CR-related predictive signature for HNSC patients, we investigated bulk RNA sequencing (RNA-seq) profiles from The Cancer Genome Atlas (TCGA) and Gene Expression Omnibus (GEO) to provide an applicable gene signature for predicting the prognosis of HNSC patients.

## Materials and methods

### Data acquisition

Gene expression profiling and clinical information for 493 HNSC patients from TCGA were obtained from UCSC Xena on July 1, 2022 (https://xena.ucsc.edu/). Notably, there are cancerous samples and paracancerous normal samples of the HNSC patients from TCGA. In the present study, to extract potentially qualified genes to establish a CR-related gene signature for predicting survival in HNSC, we only included the cancerous samples of HNSC patients, and thus the gene expression profiles used for the downstream analysis were all from cancerous samples. Among 493 HNSC patients, 112 HNSC patients had a clear HPV status, including 34 HPV+ and 78 HPV- patients with HNSC. The microarray RNA-seq data and survival information of 76 HNSC patients were obtained from the GSE41613 dataset in GEO. The microarray expression data and the corresponding disease-free survival (DFS) data of 109 HNSC patients were obtained from the GSE27020 cohort in GEO. The GSE41613 dataset was based on the GPL570 platform (Affymetrix Human Genome U133 Plus 2.0 Array), and the GS27020 dataset was based on the GPL96 platform (Affymetrix Human Genome U133A Array). RNA-seq data from TCGA and GSE27020 were normalized in the form of transcripts per million values and then log2 (*x* + 1)-transformed. The somatic nucleotide variation (SNV) data of HNSC patients were obtained from TCGA database. As discussed previously, the SNV data were also from the cancerous samples of HNCS patients from TCGA. Detailed information for the datasets is shown in **Table 1**. There were 84 circadian rhythm-related genes, which come from the molecular signature database (15), used to select qualified candidate genes in the present study (**Supplementary Table S1**).

**Table 1:** Sample information in the datasets.

Database	Normalization method	Sample number with survival information	Survival time type	HPV status
Training set	TPM	345	OS	23 HPV+, 57 HPV-
Validation set	TPM	148	OS	11 HPV+, 21 HPV-
GSE41613	gcRMA algorithm	76	OS	76 HPV -
GSE27020	TPM	109	DFS	Unknown

TPM, transcripts per million; RMA, robust multiarray analysis; OS, overall survival; DFS, disease-free survival.

### Estimation of enrichment scores for individual patients

To quantify the expression levels of the CR gene set in individual patients, we estimated the enrichment score (ES) of the CR-related gene set for individual HNSC patients using single-sample gene set enrichment analysis (ssGSEA) (16). ssGSEA is a mathematical methodology to estimate the relative expression levels of a given gene set using RNA-seq data. The parameters used in this study were as follows: min.sz = 1, max.sz = Inf, and tau = 0.25, where min.sz represents the minimum size of the resulting gene sets, max.zs represents the maximum size of the resulting gene sets, and tau represents the exponent defining the weight of the tail in the random walk performed by ssGSEA.

### Construction of the gene signature

A total of 493 HNSC patients from TCGA were randomly divided into the training set (*n* = 345) and validation set (*n* = 148). We assigned a number to each of the 493 patients, ranging from 1 to 493. Then, we randomly selected 70% of the patients as the training set based on a random sampling method that can be performed using the ‘sample’ function in R. Then, the rest of the patients were considered as the validation set. The CR-associated genes were first screened for eligible genes to establish the predictive signature using univariate Cox regression and then further analyzed using least absolute shrinkage and selection operator (LASSO) regression. The eligible genes in LASSO were utilized to construct a gene signature based on the expression levels of the eligible genes and their corresponding coefficients in LASSO using the following formula: *PER2* × (-0.2371) + *PER3* × (-0.0807) + *GHRL* × (-0.2003) + *CSF2* × (0.0277) + *HDAC3* × (0.5065) + *KLF10* × (0.1196) + *PRKAA2* × (0.2072) + *PTGDS* × (-0.0698) + *RORB* × (-0.1509).

### Assessment of the predictive performance of the gene signature

The predictive ability of the gene signature was assessed by two analyses, namely, the receiver operating characteristic (ROC) curve and Kaplan‒Meier curve, based on the risk score calculated using the abovementioned formula. The area under the curve (AUC) and log-rank test were performed in the training set, the validation set, and an independent test set. Notably, the published signatures that were used to compare with our gene signature have their own formulas for estimating the risk score, which are available in the corresponding published papers. Thus, we calculated the risk score based on the gene mRNA expression levels and the coefficients that were generated in each study.

### Functional enrichment analysis

Functional enrichment analysis was performed using the “clusterProfiler” package in R (version: 3.18.1) (17), which can determine whether canonical biological processes and signaling pathways are significantly enriched in a given patient cohort based on gene expression profiles. Information on canonical biological functions and signaling pathways is available in the GO.db and KEGG.db Bioconductor annotation data.

### Statistics

Statistical analysis was performed using R software (Version 4.0.1). The optimal cutoff value was estimated using the ‘surv_cutpoint’ function in “survival” package in R. Independent-sample *t*-tests or Wilcoxon signed rank tests were utilized according to the homogeneity of variance and normal distribution of data. Spearman’s correlation coefficient was utilized to investigate the relationship between two continuous variables. Statistical significance was considered when the *P*-value was less than 0.05.

## Results

### Construction of a circadian rhythm-related gene signature

**Figure 1:**
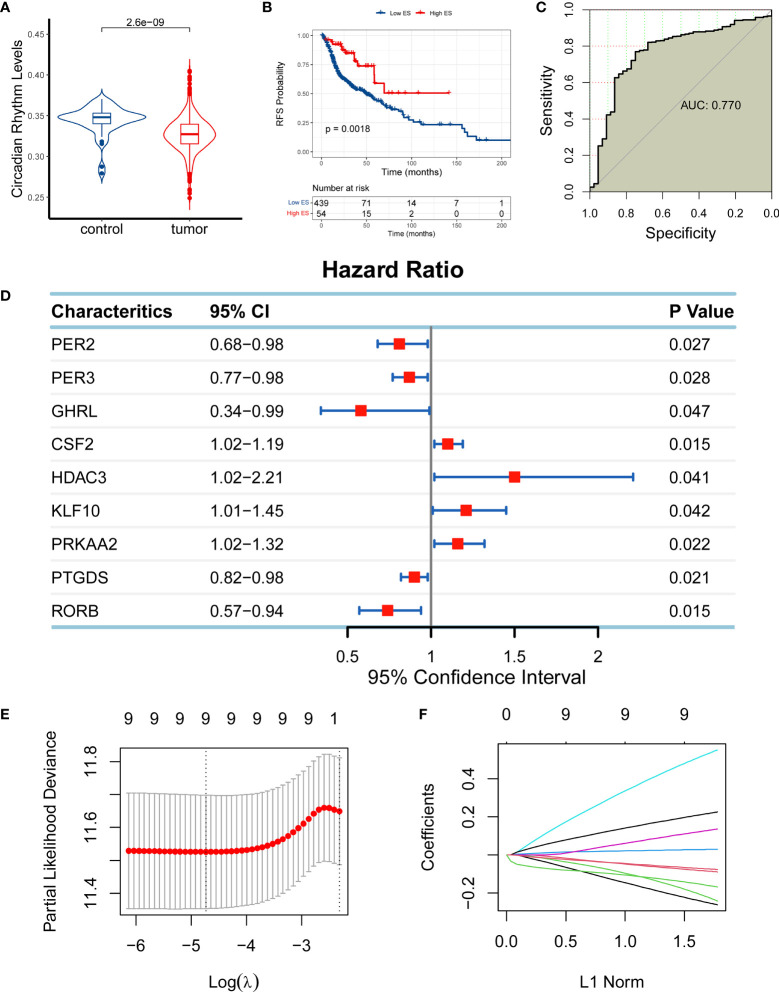
Establishment of a circadian rhythm (CR)-related gene signature in head and neck squamous cell carcinoma (HNSC). **(A)** The enrichment score (ES) of the CR-related gene set was significantly reduced in HNSC samples compared with normal samples adjacent to the cancer. **(B)** High-ES patients had an improved overall survival (OS) compared with their counterparts. **(C)** The CR-related gene set was positively enriched in normal tissue compared with tumor tissue. **(D)** Nine CR-related genes qualified for univariate Cox regression analysis (*PER2*, *PER3*, *GHRL*, *CSF2*, *HDAC3*, *KLF10*, *PRKAA2*, *PTGDS*, and *RORB*; *P* < 0.05). **(E, F)** Nine qualified genes were further validated using least absolute shrinkage and selection operator regression analysis to eliminate multicollinearity for the establishment of a CR-related gene signature for predicting OS in HNSC patients.

CR has been reported to play a role in cancer; however, it remains unclear whether it has an effect on HNSC. To investigate its association with HNSC, we compared the expression levels of circadian rhythm genes between HNSC and normal tissues using RNA-seq data from HNSC patients from TCGA cohort. The expression levels of the circadian rhythm signaling pathway were quantified as an ES using the ssGSEA algorithm based on the RNA-seq data of 493 HNSC samples from TCGA cohort. The findings showed that the circadian rhythm gene set was significantly reduced in HNSC samples compared with normal samples adjacent to the cancer (**Figure 1A**). Moreover, we divided the HNSC patients into low-ES and high-ES groups according to the optimal cutoff value of ES and performed a survival analysis. High-ES patients had an improved overall survival (OS) compared with low-ES patients (**Figure 1B**). Moreover, circadian rhythm levels accurately discriminated tumor patients from normal patients with an area under the curve of 0.770 (**Figure 1C**). These findings suggested that circadian rhythm is correlated with HNSC.

Based on the relationship between CR and HNSC, we next investigated if CR can predict the prognosis of HNSC patients by developing a CR-related gene signature to predict the survival of HNSC patients. To identify eligible CR-related genes in HNSC, we first performed a univariate Cox regression analysis for 84 CR-related genes, and we obtained nine genes (*PER2*, *PER3*, *GHRL*, *CSF2*, *HDAC3*, *KLF10*, *PRKAA2*, *PTGDS*, and *RORB*; *P* < 0.05; **Figure 1D**). The nine genes were further filtered using LASSO regression analysis to eliminate multicollinearity, which indicated that all nine genes (*PER2*, *PER3*, *GHRL*, *CSF2*, *HDAC3*, *KLF10*, *PRKAA2*, *PTGDS*, and *RORB*) could be used for the establishment of a CR-related gene signature for predicting OS in HNSC patients (**Figures 1E, F**). The CR-related gene signature was quantified based on the mRNA expression levels and the corresponding coefficients of seven CR-related genes using the following formula: *PER2* × (-0.2371) + *PER3* × (-0.0807) + *GHRL* × (-0.2003) + *CSF2* × (0.0277) + *HDAC3* × (0.5065) + *KLF10* × (0.1196) + *PRKAA2* × (0.2072) + *PTGDS* × (-0.0698) + *RORB* × (-0.1509). The coefficients represented the influence of genes on OS, in which positive coefficients represented a risk factor for OS and negative coefficients represented a protective factor for OS.

### Assessment of predicting the performance of the CR-related gene signature

**Figure 2:**
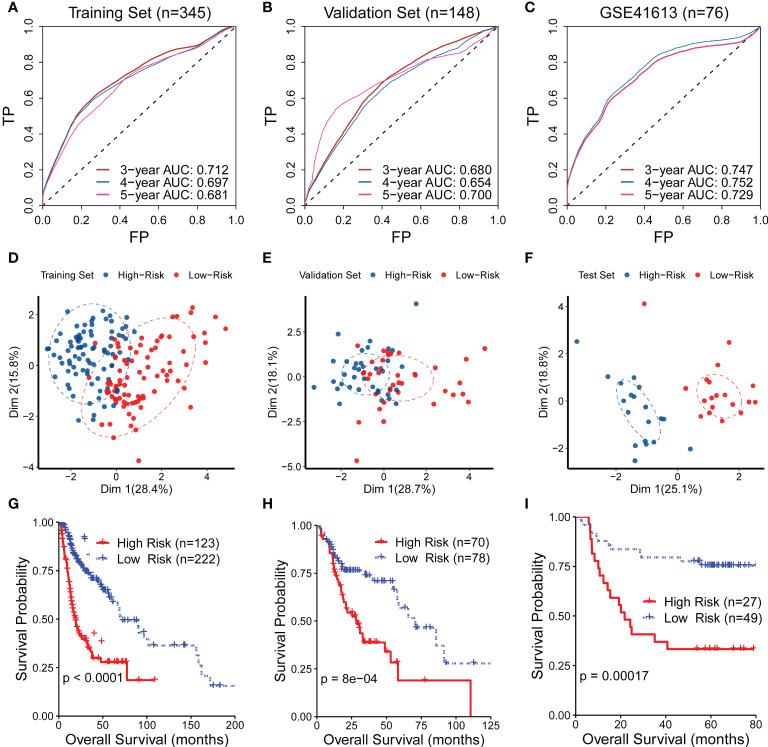
Evaluation of the performance of the circadian rhythm-related gene signature. **(A–C)** The area under the receiver operating characteristic curve (AUC) values for predicting the 5-year overall survival (OS) were 0.681, 0.700, and 0.729 in the training set, validation set, and test set, respectively. **(D–F)** The principal component analysis showed that the low-risk patients were distinct from the high-risk patients in Dim 1 in the training set, validation set, and test set. **(G–I)** The survival analysis also revealed that the low-risk group had an improved OS compared with the high-risk group in the training set, validation set, and external test set.

The predictive capability of the CR-related gene signature was assessed using ROC curves in the training set (*n* = 345), the validation set (*n* = 148), and the external test set (GSE41613; *n* = 76). The AUC values for predicting 5-year overall survival were 0.681, 0.700, and 0.729 in the training set, validation set, and test set, respectively (**Figures 2A–C**), suggesting its ability to predict OS. We then quantified the CR-related gene signature using the abovementioned formula and divided the patients into low- and high-risk groups according to the median risk score. The principal component analysis showed that the low-risk patients were distinct from the high-risk patients in Dim 1, suggesting a discriminative ability of the CR-related gene signature (**Figures 2D–F**). Consistently, the survival analysis also revealed that the low-risk group had an improved OS compared with the high-risk group in the training set, validation set, and external test set (log-rank test, *P* < 0.001; **Figures 2G–I**). These results indicated that the mRNA levels of the critical genes could reflect prognosis, which is consistent with the previous study that the pivotal genes with the highest survival scores could be used as predictive and prognostic biomarkers (18). Collectively, these findings confirmed that the CR-related gene signature has a predictive capability for OS in HNSC.

We also compared the performance of the CR-related gene signature to other published signatures using ROC curves and Kaplan–Meier curves in the validation set, training set, and external test set. The CR-related gene signature exhibited the highest AUC value among the tested signatures (**Supplementary Figures S1A, B**). The other three published signatures showed a discriminating ability in the Kaplan‒Meier curve with a significantly improved survival in the low-risk group compared with the high-risk group (**Supplementary Figures S1C, E**). We next investigated the statistical significance of the performance improvement for the CR-based signature compared with the other reported gene signatures. To this end, we compared the performance of these gene signatures 100 times using 80% of HNSC patients randomly sampled from the test set, and we compared the 100 AUC values of each signature to determine whether there was a significant difference. The results demonstrated that there was a significant distinction as shown in **Supplementary Figure S1F**.

### Comparison of the predictive capability of the CR-related gene signature with other indicators

**Figure 3:**
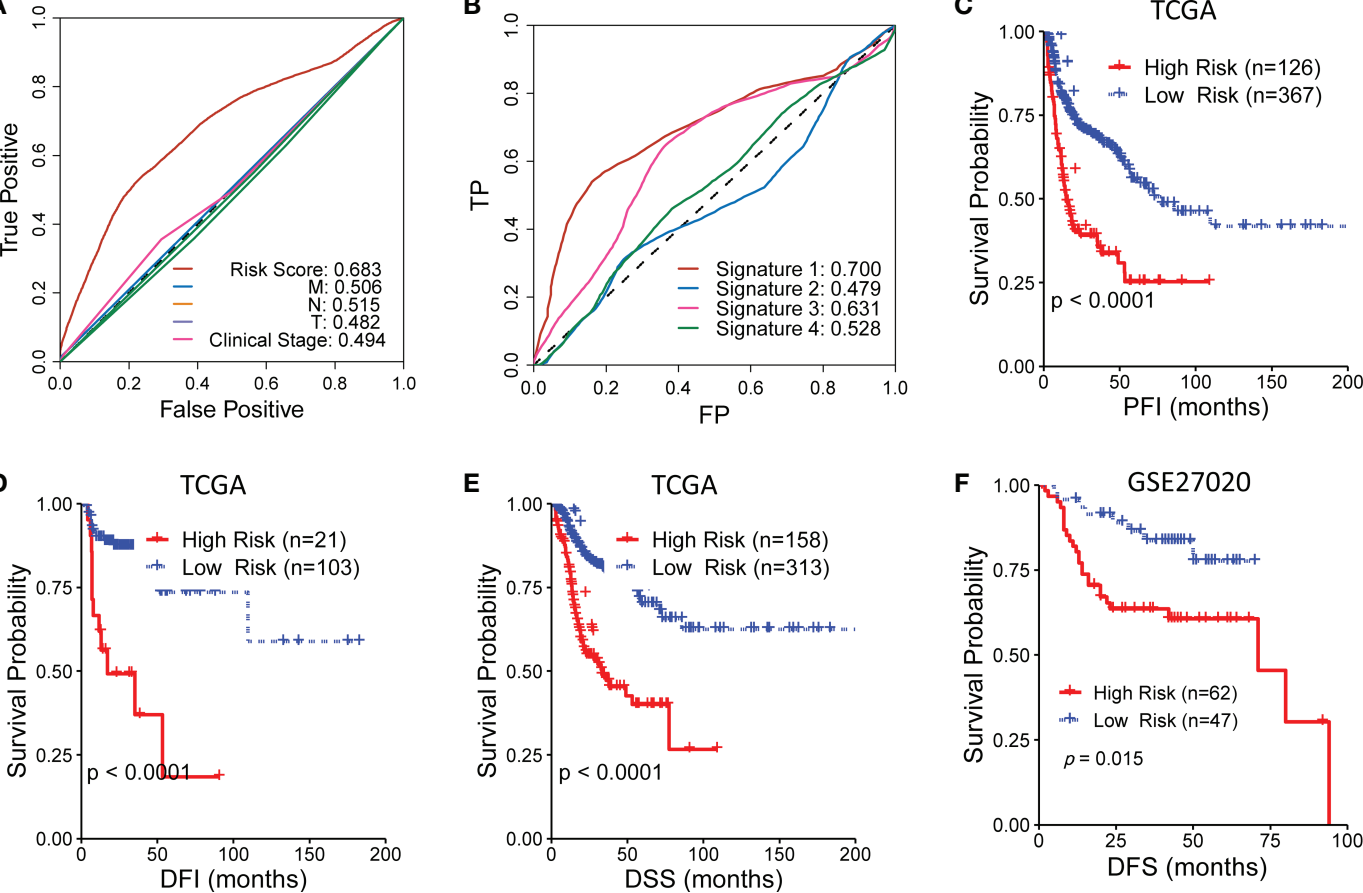
Comparison of the circadian rhythm (CR)-related gene signature with other indicators for overall survival in head and neck squamous cell carcinoma. **(A)** The CR-related gene signature showed an improved predictive performance compared with clinical T staging, clinical N staging, clinical M staging, and clinical stage. **(B)** The CR-related gene signature (Signature 1) also showed an improved predictive performance compared with the three reported gene signatures, namely, an immune-related gene signature (5) (Signature 2), an eight-gene signature (4) (Signature 3), and an autophagy-related gene signature (3) (Signature 4). **(C–E)** The low-risk group had a significantly improved progression-free interval, disease-free interval, and disease-specific survival compared with the high-risk group in The Cancer Genome Atlas. **(F)** The low-risk group had a significantly improved disease-free survival compared with the high-risk group in GSE27020.

To further evaluate the predictive capability of the CR-related gene, we compared the predictive capability of the CR-related gene signature with other indicators of OS, including clinical characteristics and three other reported gene signatures. The results showed that the CR-related gene signature showed an improved predictive performance compared with other clinical indicators (clinical T staging, clinical N staging, clinical M staging, and clinical stage) with a maximum AUC value of 0.683 (**Figure 3A**). Actually, the relationship of the signature with TNM staging had been verified in an earlier study where many kinases have expressions that correlate with T, N, and M staging (18). Moreover, the CR-related gene signature (Signature 1) also showed an improved predictive performance compared with the other three reported gene signatures, namely, an immune-related gene signature (5) (Signature 2), an eight-gene signature (4) (Signature 3), and an autophagy-related gene signature (3) (Signature 4), with AUC values of 0.700 *vs*. 0.479, 0.631, and 0.528, respectively (**Figure 3B**). Collectively, these findings indicated that the CR-related gene signature has a better predictive ability than the other gene signatures.

Furthermore, we assessed the ability of the CR-related gene signature to predict the progression-free interval (PFI), disease-free interval (DFI), and disease-specific survival (DSS) in TCGA cohort. Surprisingly, the low-risk group had a significantly improved PFI, DFI, and DSS compared with the high-risk group (log-rank test, *P* < 0.001; **Figures 3C–E**). Moreover, the low-risk group had a significantly better DFS compared with the high-risk group in another independent cohort (GSE27020; log-rank test, *P* = 0.015; **Figure 3F**). These results further supported the acceptable prognostic ability of the CR-related gene signature in HNSC.

### Clinical significance of the CR-related gene signature

**Figure 4:**
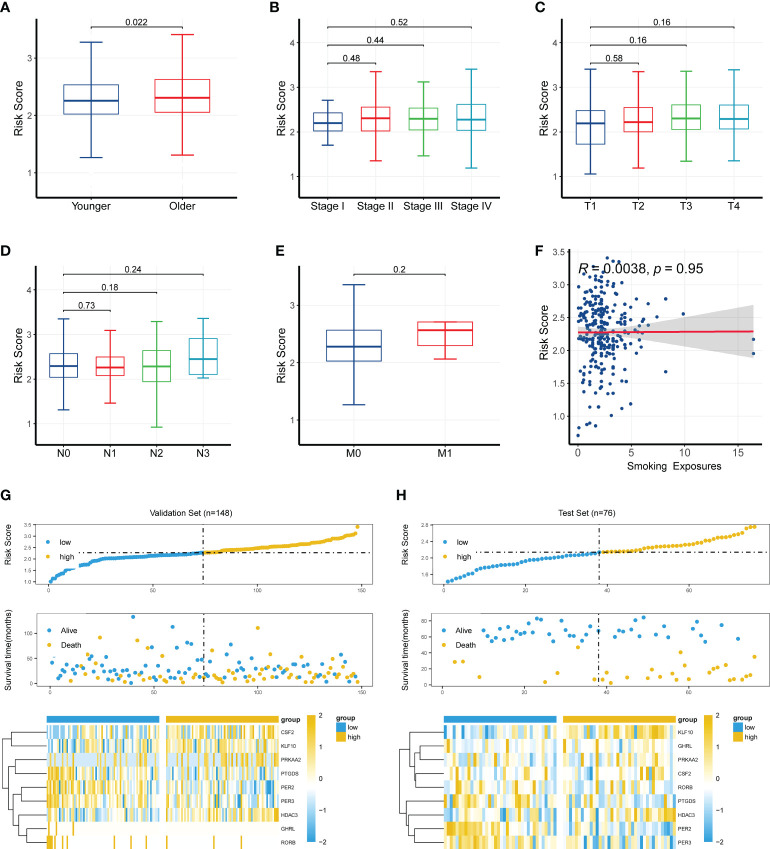
Clinical significance of the circadian rhythm-related gene signature. **(A)** Older patients had a higher risk score than younger patients (cutoff value of 60 years old; *P* < 0.05). **(B–F)** The risk score was not related to clinical stage, TNM staging, or smoking exposure. **(G)** A higher risk score was associated with a higher mortality and a higher expression level of CSF2, KLF10, PRKAA2, and HDAC3 in the training set. **(H)** A higher risk score was associated with a higher mortality and a higher expression level of CSF2, KLF10, PRKAA2, and HDAC3 in the validation set.

To further investigate the clinical significance of the CR-related gene signature, we analyzed the relationship between the CR-related gene signature and clinical characteristics. We found that the older patients had a higher risk score than the younger patients (cutoff value of 60 years old; *P* < 0.05; **Figure 4A**), whereas the risk score was not related to clinical stage, TNM staging, and smoking exposure (**Figures 4B–F**). Based on the median value of 61 years old, the cutoff value was 60 years old; to facilitate practical clinical application, we artificially positioned the threshold to 60 years old. We next explored the relationship of the risk score with survival status and the expression levels of the nine genes. The findings showed that higher risk scores were associated with higher mortality and higher expression levels of CSF2, KLF10, PRKAA2, and HDAC3 in the training set (**Figure 4G**) and validation set (**Figure 4H**).

Furthermore, we analyzed the relationship between CR-related gene expression and age. Except for *PER3*, no other genes were significantly changed between the younger and older groups (**Supplementary Figures S2A–I**). However, different proportions of HPV+ status were present in different age groups, which may have influenced the risk score and performance of the CR-related gene signature (**Supplementary Figures S2J–L**).

### Functional enrichment analysis

**Figure 5:**
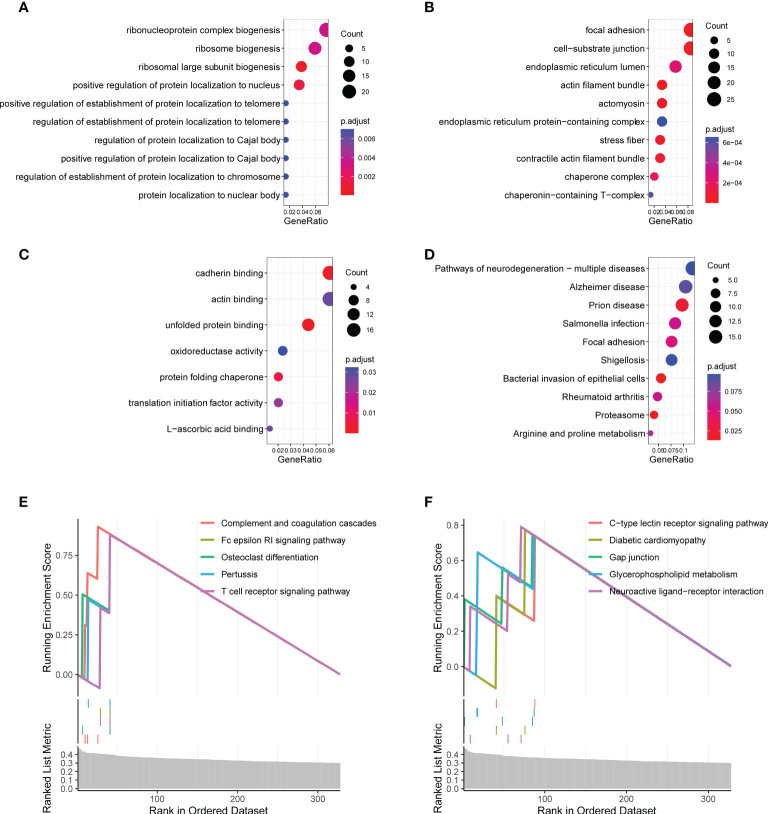
Functional enrichment analysis of the circadian rhythm-related gene signature. **(A)** Enriched biological processes included ribonucleoprotein complex biogenesis, ribosome biogenesis, and ribosomal large subunit biogenesis. **(B)** Enriched cell components included focal adhesion, cell–substrate junction, endoplasmic reticulum lumen, and actin filament bundle. **(C)** Enriched molecular functions included cadherin binding, actin binding, and unfolded protein binding. **(D)** Enriched Kyoto Encyclopedia of Genes and Genomes (KEGG) pathways included focal adhesion, proteasome, arginine, and proline metabolism. **(E, F)** The gene set enrichment analysis showed the top 10 KEGG signaling pathways, including complement and coagulation cascades as well as the Fc epsilon RI signaling pathway and T cell receptor signaling pathway.

To investigate the biological functions associated with the CR-related gene signature, we performed functional enrichment analysis for genes that were correlated with the CR-related gene signature. We calculated the correlation coefficients between the gene signature and all genes, which included 405 qualified genes (*P* < 0.01, *R* > 0.3) (19). The analysis using the “clusterProfiler” package in R indicated that these genes were mainly enriched in ribosome biogenesis, focal adhesion, and protein localization (**Figures 5A–D**; **Supplementary Table S2**). The gene set enrichment analysis showed that some enriched biological functions were associated with immune functions, including complement and coagulation cascades as well as the Fc epsilon RI signaling pathway and the T cell receptor signaling pathway (**Figures 5E, F**).

### Association of the CR-related gene signature with the tumor immune microenvironment

**Figure 6:**
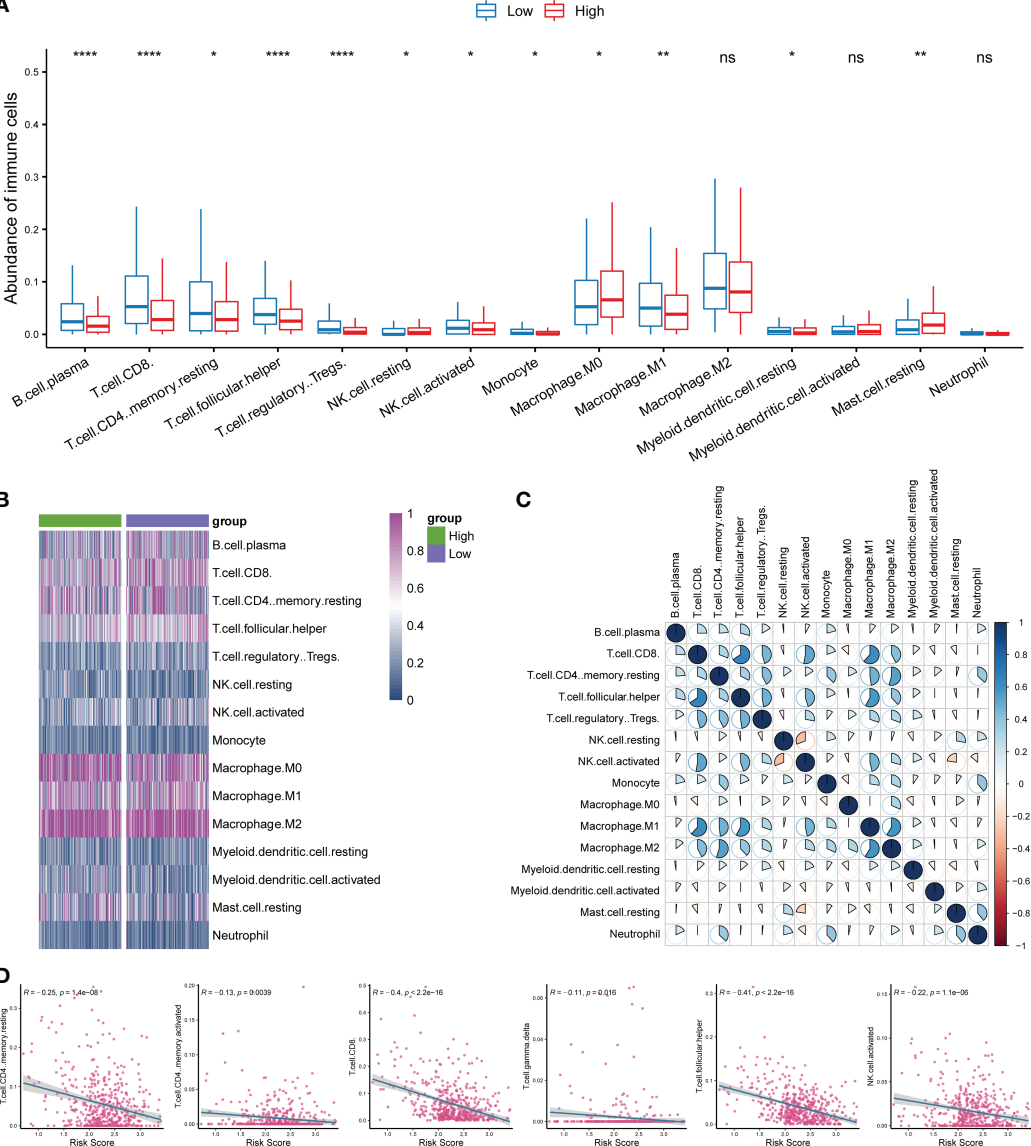
Investigation of the correlation of the immune microenvironment with circadian rhythm. **(A)** Comparison of the abundance of immune cells between the high- and low-risk groups. **(B)** The heat map analysis indicated a distinct expression of tumor-infiltrating immune cells between groups. **(C)** Activated NK cells were significantly positively correlated with CD8+ T cells, T cell follicular helper cells, and M1 macrophages. **(D)** The risk score was negatively correlated with CD4^+^ T cells, CD8^+^ T cells, gamma delta T cells, and activated NK cells. *P < 0.05, **P < 0.01, and ***P < 0.001.

Considering that the functional enrichment analysis implicated immune function in HNSC, we analyzed the changes in the abundance of immune cells between the high- and low-risk groups using bulk RNA-seq data. The abundance of immune cells was estimated using the CIBERSORT algorithm. The results showed that B cell plasma, CD8^+^ T cells, memory resting CD4^+^ T cells, T cell follicular helper cells, regulatory T cells, activated NK cells, monocytes, and M1 macrophages were significantly upregulated in the low-risk group (*P* < 0.05; **Figure 6A**), whereas resting NK cells, M0 macrophages, and resting mast cells were significantly upregulated in the high-risk group (*P* < 0.05; **Figure 6A**). The heat map analysis also indicated a distinct expression of these cells between groups (**Figure 6B**). The correlation analysis showed that activated NK cells were significantly positively correlated with CD8 T cells, T cell follicular helper cells, and M1 macrophages (**Figure 6C**). Consistently, we found that the risk score was negatively correlated with CD4^+^ T cells, CD8^+^ T cells, gamma delta T cells, and activated NK cells (**Figure 6D**; *P* < 0.05).

### Profiling of somatic nucleotide variation in HNSC patients between the low- and high-risk groups

**Figure 7:**
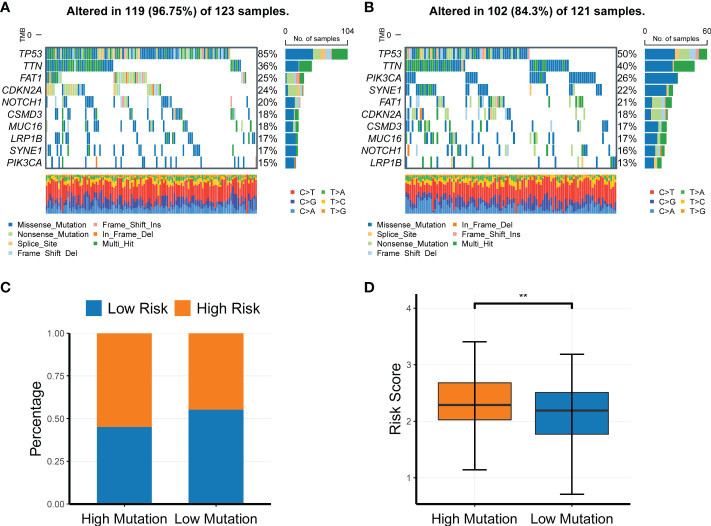
Profiling of somatic nucleotide variation for head and neck squamous cell carcinoma patients of different risk groups. **(A, B)** Waterfall plot of the nucleotide variation rate in the high-risk group and the low-risk group. **(C, D)** Bar plots and box plots of risk scores in the high-mutation group and low-mutation group. **P < 0.01.

To investigate the association of the CR-related gene signature with the mutation levels in HNSC patients, we profiled the mutation landscape of the low-risk and high-risk groups by analyzing the somatic nucleotide variation data of HNSC patients from TCGA cohort using the “maftool” package in R. To better compare the potential distinction of the mutation landscape between different risk score groups, we defined the patients with the top 25% risk score as the high-risk group, and we defined the patients with the bottom 25% risk score as the low-risk group. The waterfall plot demonstrated that the high-risk group had a higher nucleotide variation rate than the low-risk group (96.75% *vs*. 84.30%, **Figures 7A, B**). Consistent with the findings of the waterfall plot, the bar plot showed that the risk score was significantly increased in the high-mutation group compared with the low-mutation group (**Figure 7C**), and the box plot also demonstrated that the risk score was significantly increased in the high-mutation group compared with the low-mutation group (**Figure 7D**).

### Effects of the CR-related gene signature on drug response

**Figure 8:**
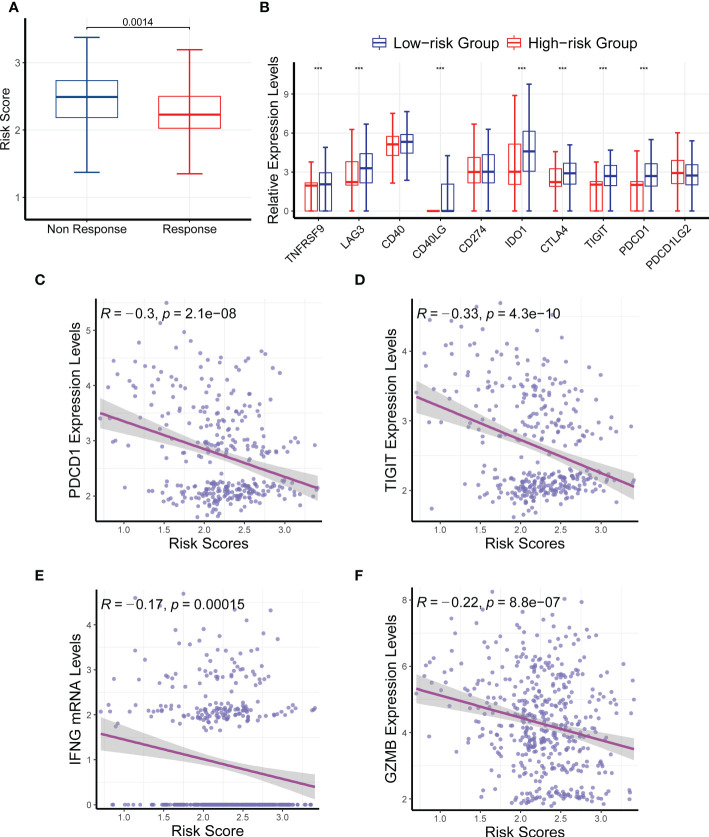
Effects of the circadian rhythm-related gene signature on drug response. **(A)** The nonresponders had a significantly higher risk score than the responders in The Cancer Genome Atlas. **(B)** The mRNA levels of *TNFRSF9*, *LAG3*, *CD40LG*, *IDO1*, *CTLA4*, *TIGIT*, and *PDCD1* were significantly upregulated in the low-risk group compared with the high-risk group. **(C–F)** The risk score was significantly correlated with the expression levels of *PDCD1*, *TIGIT*, *INFG*, and *GZMB*. ***P < 0.001.

Because the gene signature was associated with the tumor immune microenvironment and somatic nucleotide variation in HNSC, we next investigated its relationship with drug response. We compared the risk scores between responders and nonresponders in TCGA and found that nonresponders had a significantly higher risk score than responders (**Figure 8A**; *P* = 0.0014). We then compared the mRNA levels of multiple immune checkpoint genes between the high- and low-risk groups. Consistently, we found that the mRNA levels of *TNFRSF9*, *LAG3*, *CD40LG*, *IDO1*, *CTLA4*, *TIGIT*, and *PDCD1* were all significantly upregulated in the low-risk group compared with the high-risk group (**Figure 8B**), further verifying the ability of the CR-related risk score to predict drug response. In addition, we compared the expression levels of more immune checkpoint molecules between the low- and high-risk groups. We retrieved 59 immune checkpoint genes from previous literature (20–23), and we found that 42 of these were differentially expressed between the low- and high-risk groups (**Supplementary Table S3**).

We also analyzed the correlation between the risk score and several immune checkpoint genes, and we found that the risk score was significantly correlated with the expression levels of *PDCD1*, *TIGIT*, *INFG*, and *GZMB* (**Figures 8C–F**; *P* < 0.05). Collectively, these findings further confirmed the ability of the CR-related gene signature to predict drug response to immune checkpoint inhibitors in HNSC.

## Discussion

We developed and validated a nine-gene CR-related signature for predicting prognosis in patients with HNSC, which may serve as a precision medicine tool. Several immune-related biological processes were enriched in HNSC, including complement and coagulation cascades as well as the Fc epsilon RI signaling pathway and the T cell receptor signaling pathway. Moreover, we observed that the CR-related gene signature was associated with somatic nucleotide variation and drug response in HNSC. Overall, these findings provide a tool for predicting prognosis and drug response in patients with HNSC, which will help to develop precision medicine.

One of the main contributions of this study is the establishment of a CR-related prognostic signature for predicting the survival of patients with HNSC. The performance of the signature was verified in an external dataset (GSE41613), which indicated that the gene signature is highly reliable and widely applicable. The predictive power of this gene signature is superior to common clinical characteristics and several reported gene signatures for predicting the 5-year overall survival in HNSC patients. Surprisingly, the maximum AUC value of the CR-based model was not consistent between comparisons, which may be due to missing clinical data points or inconsistent baselines across populations. Specifically, we investigated the optimal performing test set and observed that it consisted of all HPV-negative patients. A subsequent analysis demonstrated that the CR-related gene signature performed better in the HPV-negative cohort. In addition, we observed that most clinical features of the CR-related gene signature were not different, and only a few clinical features were significantly different, which may be due to an unbalanced sample size between primary and metastatic patients (491 *vs*. 2) or some commonly used clinical features, such as N staging, may not be an ideal predictor for risk. Further investigation of this aspect is warranted.

Another important finding was that several important biological processes were identified to be involved in CR and high risk as follows: AM immune function, complement and coagulation cascades, Fc epsilon RI signaling pathway, and T cell receptor signaling pathway. Consistent with our findings, complement and coagulation cascades have been reported to be involved in HNSC (24). We also showed that the T cell receptor signaling pathway was enriched in the high-risk group. Similarly, a pancancer analysis revealed that disrupted circadian rhythm is associated with T cell exhaustion (25), and the response of T cells to antigens has a circadian variation (26, 27). Here we found that the CR-related gene signature was associated with these immune-related signaling pathways, suggesting the role of circadian rhythm disruption in the tumor immune microenvironment.

The nine genes comprising the CR-related gene signature may be potential biomarkers for HNSC. The dysfunction of *PER2* and *PER3* has been related to cancer development and progression (28–30) as well as poor prognosis in HNSC (31). In addition, high levels of *KLF10* are associated with a favorable prognosis in patients with HNSC (32). Colony-stimulating factor 2 (*CSF2*) plays an important role in macrophage polarization (33) and may be associated with poor prognosis in breast cancer and colorectal cancer (34, 35). However, further investigations on the role of *GHRL* and *RORB* in HNSC are warranted.

The present study has important implications for the treatment and prognosis of HNSC. First, our study provided a novel prognostic signature that may aid clinical treatment strategies for HNSC. Second, we revealed several critical oncogenes and pathways that may serve as promising therapeutic targets for the treatment of HNSC. Nevertheless, further *in vitro* and *in vivo* investigations are warranted to study the role of these pivotal genes in HNSC and their precise mechanisms of action.

The present study had several limitations that warrant further research. First, the key genes and signaling pathways in this study were identified using bioinformatics analysis, and further *in vitro* and *in vivo* studies are required to explore their physiological mechanisms of action. In addition, the risk score was identified to predict the drug response of HNSC, warranting further clinical investigation.

In conclusion, we successfully constructed and validated a novel CR-related signature that predicts the prognosis of patients with HNSC, thereby providing a rationale for the further investigation of HNSC.

## Data availability statement

The original contributions presented in the study are included in the article/**Supplementary Material**. Further inquiries can be directed to the corresponding author.

## Author contributions

CZ and DD collected the data. MY, CZ, and DD analyzed the data and wrote the manuscript. HW, JD and SS contributed to manuscript revision. All authors contributed to the article and approved the submitted version.

## Funding

This project was supported by the National Natural Science Foundation of China (grant numbers. 81902111); the Natural Science Foundation of Jilin Province (grant numbers. 20220101281JC).

## Conflict of interest

The authors declare that the research was conducted in the absence of any commercial or financial relationships that could be construed as a potential conflict of interest.

## Publisher’s note

All claims expressed in this article are solely those of the authors and do not necessarily represent those of their affiliated organizations, or those of the publisher, the editors and the reviewers. Any product that may be evaluated in this article, or claim that may be made by its manufacturer, is not guaranteed or endorsed by the publisher.
