# Supplementary figures and images for: Acircadian rhythm-related gene signature for predicting survival and drug response in HNSC
Source: Front Immunol. 2022 Nov 24;13:1029676. doi: 10.3389/fimmu.2022.1029676 (PMC9729285; doi:10.3389/fimmu.2022.1029676)

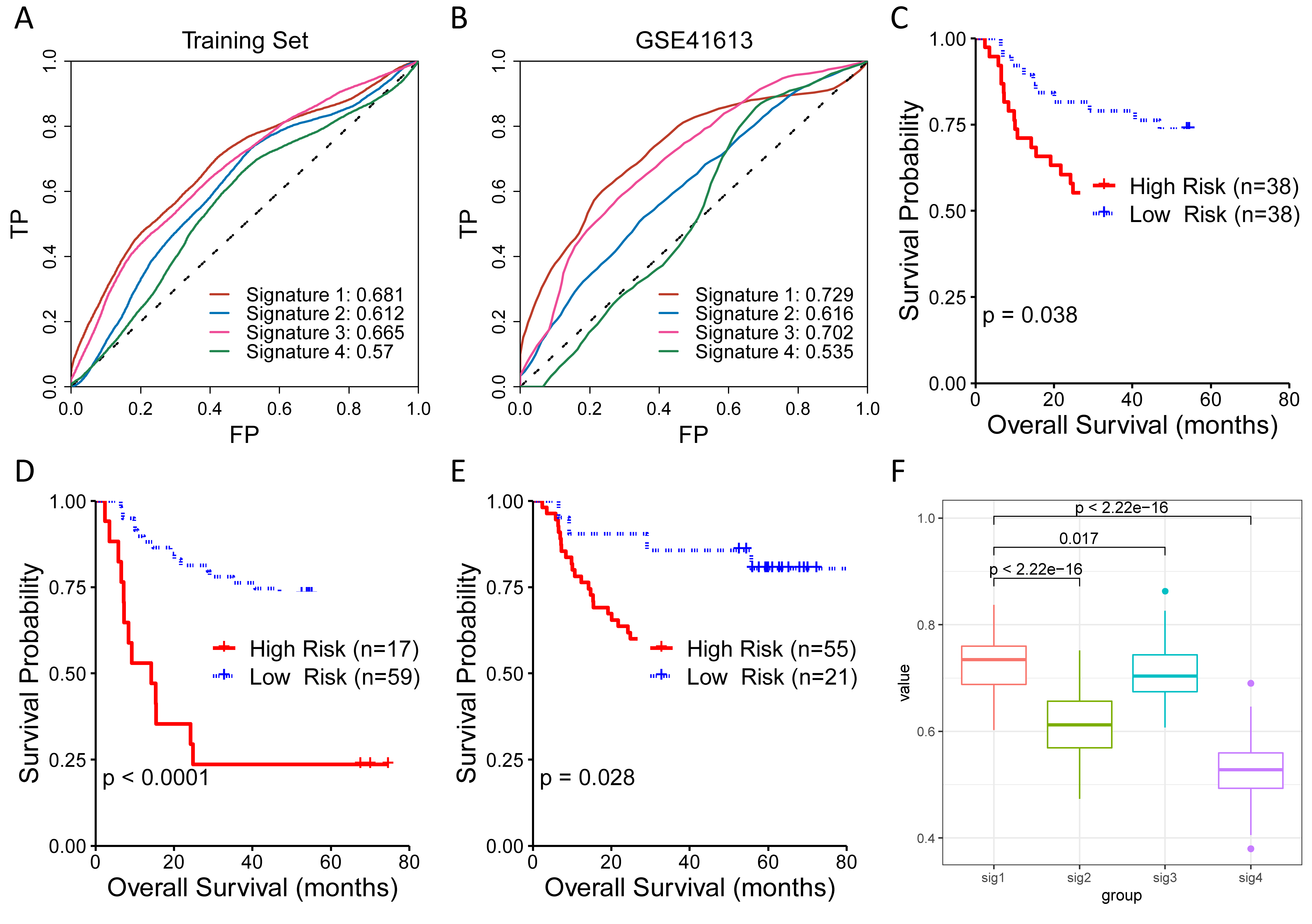

Supplement: Supplementary file 1 [file Image_1.tif]

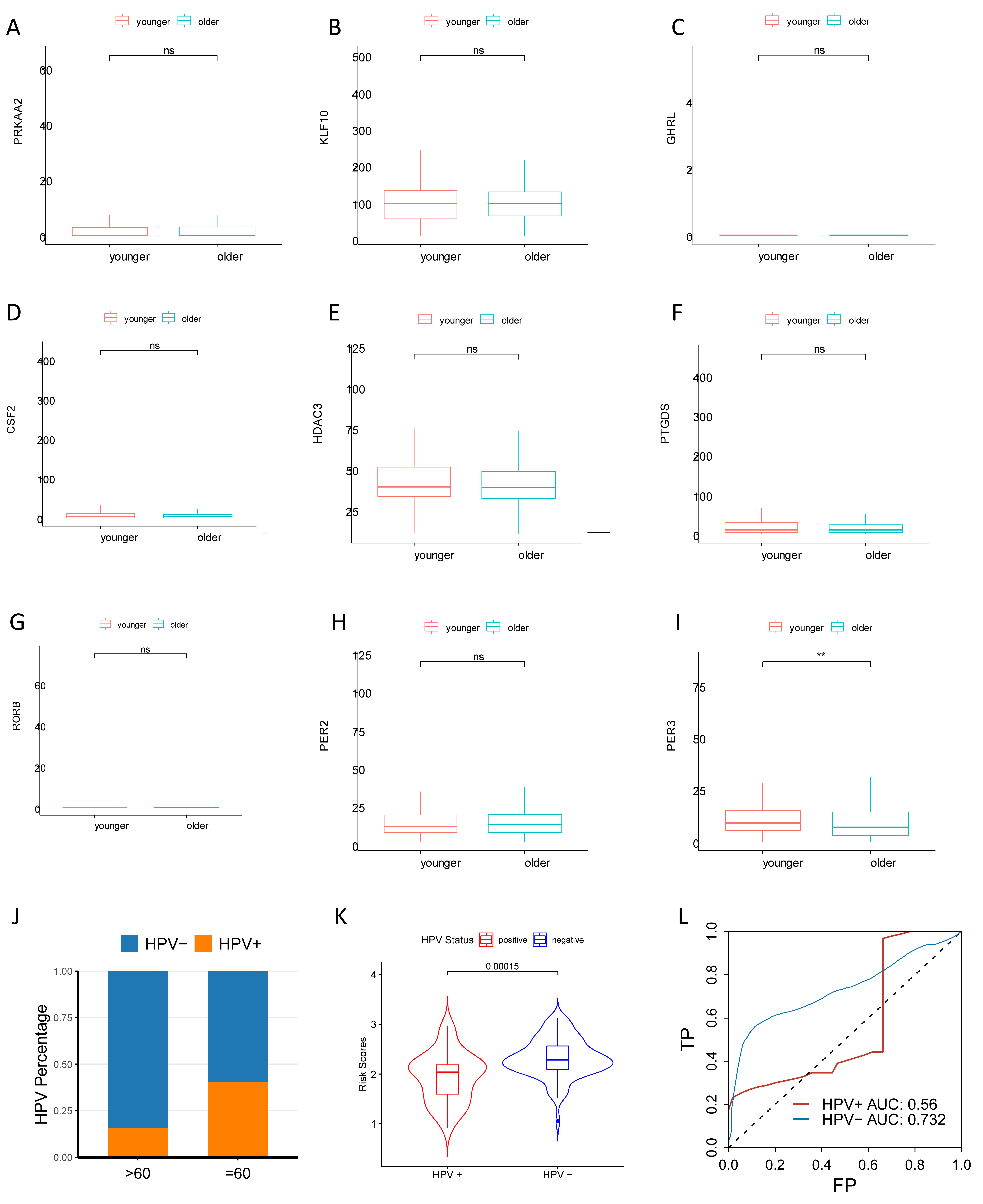

Supplement: Supplementary file 2 [file Image_2.tif]
